# Indelible Ink

**Published:** 1870-12

**Authors:** 


### INDELIBLE INK.

The common kind is made of nitrate of silver, and stains the skin; another kind is made of the Anacardia nut, is of a brown color, and has a smell of ether. It is a virulent poison, causing inflammations and eruptions wherever it touches the skin. The nitrate of silver has no odor.

